# The Advances in Molecular and New Point-of-Care (POC) Diagnosis of Schistosomiasis Pre- and Post-praziquantel Use: In the Pursuit of More Reliable Approaches for Low Endemic and Non-endemic Areas

**DOI:** 10.3389/fimmu.2019.00858

**Published:** 2019-05-28

**Authors:** Marta G. Cavalcanti, Aline Fernandes Araujo Cunha, José Mauro Peralta

**Affiliations:** ^1^Serviço de Doenças Infecciosas e Parasitárias, Hospital Universitário Clementino Fraga Filho, Universidade Federal do Rio de Janeiro, Rio de Janeiro, Brazil; ^2^Departmento de Imunologia, Instituto de Microbiologia Paulo de Góes, Universidade Federal do Rio de Janeiro, Rio de Janeiro, Brazil

**Keywords:** schistosomiasis, molecular diagnosis, POC-CCA, POC-CAA, praziquantel

## Abstract

Like soil-transmitted helminth infections, schistosomiasis is an important neglected tropical disease (NTD) related to poverty with a major impact on public health in developing countries. Diagnosis of active infection is crucial for surveillance of controlled or post-elimination schistosomiasis areas. In addition, the use of conventional diagnostic tools in non-exposed populations (such as travelers) results in misdiagnoses in the prepatent period of infection. Also, the accuracy of standard tests applied in low-endemicity areas (LEAs) decreases after several rounds of treatment. We aimed to determine whether it would be necessary to replace schistosomiasis conventional diagnostic tests such as parasitological methods in LEAs. Also, we evaluate the use of new tools in non-endemic areas. Reliable, cheap and easy-to-use diagnostic tools are needed to respond to the demands of a new era of elimination and eradication of schistosomiasis. To this end, molecular diagnosis—including nucleic acid-based assays (loop-mediated isothermal amplification, polymerase chain reaction) and circulating cathodic and anodic antigen detection tests have become promising strategies. In this review, we attempt to address the use of alternative diagnostic tests for active infection detection and drug-monitoring after specific schistosomiasis treatment.

## Introduction

The World Health Organization (WHO) set a high standard: eliminating schistosomiasis until the end of the decade. In 2012's, delegates in the World Health Assembly adopted WHA65.21 resolution which urged the Member States to get involved in plans with achievable targets toward elimination. Endemic countries would also ensure the provision of therapies, being WHO task to elaborate a procedure to evaluate the interruption of schistosomiasis transmission. Also, to achieve this goal, the WHO established partnerships with non-profit organizations participating in initiatives that hopefully will allow morbidity control of schistosomiasis in areas of Sub-Saharan Africa (1–4). The ultimate objective is to decrease the risk of infection in exposed populations, e.g., school-aged children, women, farmers and fishermen in areas of high prevalence. In addition, low endemic areas (LEA) try to achieve this goal: sustainable schistosomiasis control and interruption of transmission in the nearby future (3). Despite the low transmission rates and low parasitic loads, individuals living in LEA are still at risk of infection. Moreover, since even light infections bear a reasonable chance of morbidity, it is essential to increase efforts toward eliminating schistosomiasis from LEAs.

Overall, the main strategies to control or eliminate schistosomiasis transmission include preventive chemotherapy, water sanitation and hygiene (WASH), snail control, and the dissemination of information, education, and communication (IEC) (5). Although most of these approaches are feasible, elimination of schistosomiasis in every transmission area is not simple. In addition, global changes are re-shaping the world map by introducing, establishing and spreading vector-borne diseases like schistosomiasis in previously non-endemic areas (6, 7). Although schistosomiasis is a poverty-related NTD, *Schistosoma* infection was recently detected in Southern Europe. In the summer of 2013, an autochthonous schistosomiasis outbreak affected travelers from France, Germany and Italy. A hybrid of human and livestock-specific *Schistosoma* was identified as a causative agent of urogenital schistosomiasis in Corsica, a tourist destination (8).

The emergence of new endemic areas and the persistence of transmission hotspots require the strategies of control and/or elimination to be perfected. As a result, surveillance systems must be able to detect early changes in transmission patterns in endemic areas and the introduction of parasites in formerly non-endemic areas, and strategies must be designed to suppress infection spread. However, the success of schistosomiasis control or elimination in the world depends on how infection is detected.

*Schistosoma* infection is primarily determined by laboratory testing. After laboratory detection of *Schistosoma* infection, it is possible to measure the effectiveness of interventions, monitor transmission in endemic areas, detection of recent introduction of the parasite into non-endemic areas and assess the drug response at community and individual levels (9). Thus, it is necessary to have reliable, accurate, and low-cost diagnostic approaches. In LEAs, traditional tests show decreased performance and underestimate infection prevalence both pre-and post-treatment. By contrast, diagnosis of schistosomiasis in non-endemic areas is also difficult for groups of recently infected travelers and immigrants from non-transmission areas. Could changes to the conventional diagnostic approaches remedy the present scenario? The advances and the relevant perspectives in schistosomiasis diagnosis are discussed here.

## Conventional Approaches Underestimate *Schistosoma* Infection Frequency: Implications for the Reliability of Pre-Treatment Diagnoses and Efficiency of Post-Therapy Interventions

Schistosomiasis diagnosis traditionally relies on ova detection in biological specimens. Positive egg-excretion is a marker of active infection. However, the peculiar life cycle of *Schistosoma*, as well as the complexities involving tissue distribution and excretion of ova makes the diagnosis of active infection far from certain in many scenarios. Dictated by parasite loads and egg production, the host's level of infection ultimately determines the tests' success. Low parasite burden in individuals living in LEAs is associated with decreased accuracy of microscopy before and after treatment (7, 10–12). Adult worms can live for decades, but elderly parasites may have reduced egg production, interfering with egg detection. Also, acquired single gender infections or after use of chemotherapy could explain the absence of egg excretion despite the persistence of infection. A variation in egg excretion and subsequent erroneous diagnosis by an inexperienced technician can mistakenly lead to underestimation of schistosomiasis prevalence or an incorrect evaluation of post-treatment response (13, 14).

Molecular assays—including DNA and antigen detection for immature forms like schistosomula and young adult worms—could help the diagnosis of infection in the prepatent period when eggs have not yet been produced. *Schistosoma's* biological sub-products, such as schistosome antigens, can be present in the blood and excreted in the urine by a single pair of adult live parasites during very early infection (pre-patent infection) (15–19). Also, schistosome antigens may remain detectable from worm pairs that no longer produce eggs after treatment with praziquantel (PZQ). The presence of urinary antigen in the absence of eggs may indicate a single sex infection or unhealthy, unfertile female or senile worms (10, 19, 20). Fragments of *Schistosoma* nucleic acids are released during the entire parasitic life cycle in intermediate and definitive hosts (humans), but are also found in the parasites' post-mortem tissue. DNA detection assays have the potential to detect schistosomiasis at day one post-infection (21).

## The Decreased Sensitivity of Conventional Tests Changes the Efficiency of Global Schistosomiasis Surveillance for the Detection of Elimination and Leads to Underdiagnoses of Light Infections at the Individual and Community Levels

In resource-limited settings, the need to keep costs low makes the use of parasitological methods such as the Kato-Katz (K-K) technique a favored diagnostic approach in transmission areas. Requirements for K-K sample preparation are usually affordable, although a well-trained technician is necessary for correctly reading the slides and identifying and counting eggs of soil-transmitted helminths (STH) and schistosomes (22). K-K is a quantitative, highly specific method traditionally used for monitoring STH in co-endemic areas, as it also detects hookworm, whipworm, and roundworm eggs as well as schistosome eggs. However, its low sensitivity compromises evaluation after drug administration, which generates large numbers of false negative results in patients with decreased parasitic loads. Also, it is a time-consuming method and requires electricity to be available. Another limitation is the inaccuracy of K-K for diagnosing early infections prior to oviposition (11, 23, 24).

Population-based studies demonstrate that the variability of parasitological test performance can be an important issue (Table 1). In moderate–to-high endemic areas, K-K may have low sensitivities varying from 41.4–83.5%. In LEAs and/or populations with low parasite burden, the K-K technique has sensitivities that may range from 12.5 to 75% (Table 1). As a result, prevalence may be underestimated in those settings. In moderate-to-high endemicity areas, prevalence is as poor as 20.4–94.8% by using the duplicate K-K technique (Table 1). However, in LEAs, prevalence varies from 4.9 to 7.4% and mostly reflects the reference test's decreased accuracy (25–27). Hence, the results indicate that the test sensitivity varies with infection prevalence and, the last one depends highly on “reference test” accuracy (31). For example, in one study in LEA, *S. mansoni* infection prevalence determined by K-K with a single slide/sample showed a prevalence of 1.9% and a test sensitivity of 62.5%, respectively (30). By increasing the number of slides per sample and/or the number of stool samples examined, prevalence estimation also increases in both moderate-to-high and low endemic areas (25, 30).

**Table 1:** Baseline comparative prevalence and sensitivity in high-moderate and low endemicity areas based on Kato-katz test in study populations not submitted to praziquantel treatment.

**Endemic area^£^**	**Reference**	**Population** **(N^**°**^)**	**Prevalence** (%)^*^	**Sensitivity%** (CI 95%)^**^
Moderate-high	Oliveira et al. (25)	270 GP	20.4	41.4 (32.8–50.5)
	Coulibaly et al. (26)	242 pSC	23.1	47.5 (38.3–56.8)
	Lamberton et al. (27)	96 SC	94.8	83.5 (74.3–90.5)
Low	Okoyo et al. (28)	1899 GP	4.9	12.5 (9.7–15.8)
	Ferreira et al. (29)	300 GP	6.0	61.1 (38.6–79.7)
	Cavalcanti et al. (30)	108 GP	7.4	75 (34.91–96.81)

K-K, Kato-Katz test;

£ *Areas are classified according WHO; pSC, pre-school children; SC, School Children; GP, General Population*.

* Prevalence calculated based on the total results obtained by the examination of two slides/stool sample/person;

** *Sensitivity was determined on the results from the examination of at least one stool sample/person (2 slides/sample of stool)*.

Since the intensity of infection and prevalence may influence schistosomiasis conventional test sensitivity, it is critical to get better solutions toward diagnosis improvement in areas of low endemicity (2, 7). In Cavalcanti et al. (30), a study performed in LEA assessed schistosomiasis prevalence by using both conventional tests and new diagnostic tools. By using duplicate K-K with a sensitivity of 75% (CI95%: 34.91–96.81), the egg counts varied from 0 to 72 eggs per gram (epg) and the estimated prevalence was 7.41% (Table 1). The results showed that the number of individuals with no egg detection could not be assessed by traditional methods, diagnosis of “real” active schistosomiasis became an issue. The use of a molecular approach in this same area changed the prevalence estimation by demonstrating that 12.96% of the study population had an active infection. Individuals with active infection were composed of 7.41% egg excretors and 21.3% non-egg excretors as determined by K-K and molecular methods, respectively (30). The introduction of molecular methods increases detection of active infection in areas of low, moderate and high endemicity (Tables 2, 3).

**Table 2:** Prevalence and sensitivity values determined by *Schistosoma mansoni* DNA detection assays before treatment in high, moderate, and low endemicity areas.

**Endemic area** **(Prevalence)^£^**	**Method** **(Type of DNA detection assay; gene target; biological sample)**	**Reference**	**Study population** **(N^**°**^)**	**New test prevalence %** **(KK prevalence^**¶**^)**	**Sensitivity % of new test** **(CI 95%)** **(Reference test)**
High	rt-PCR (ITS2) Feces	Al-Shehri et al. (32)	258^*^ SC	67.4 (44,1)	85 (78.4–89.9) (POC-CCA)
					90.2 (84.2–96.2) (LCA)
	LAMP (121-bp tandem repeat) Feces	Mwangi et al. (33)	383 SC	45 (46)	97.2 (93.5–98.8) (K-K)
	PCR-ELISA (121-bp tandem repeat) Urine	Lodh et al.(34)	100 GP	89 (51)	100 (95–100) (K-K/PCR)
	rt-PCR (121-bp tandem repeat) Feces	Fuss et al. (35)	305 SC	92.9 (85.2)	96.8 (94.9–98.8) (K-K)
					98.7 (96.5–100) (LCA)
Moderate low	PCR-ELISA (121-bp tandem repeat) Feces	Gomes et al. (36)	206 GP	30.1 (13.1)	96.3 (81.7–99.8) (K-K)
	PCR (121-bp tandem repeat) Urine	Enk et al. (7)	194 GP	41.2 (13.9)	100 (94.7–100) (Combined 1^**^)
Low	SmMIT-LAMP (mitochondrial *S*. *mansoni* minisatellite DNA region) Feces	Gandasegui et al. (37)	427^*^ GP	30.2 (3.04)	92.9 (66.1–99.8) (K-K)
	r-t PCR (COX-1) Feces	Cavalcanti et al. (30)	108 GP	29.6 (7.4)	100 (63.1–100) (K-K)
	PCR (121-bp tandem repeat) Urine	Hessler et al. (38)	111 SC	78 (8.0)	100 (96–100) (K-K)

£ Classified according WHO; rt-PCR, Real-Time Polymerase Chain Reaction; LAMP, Loop-Mediated Isothermal Amplification Assay; SmMIT-LAMP, Sm Mitochondrial Loop-Mediated Isothermal Amplification Assay; SC, School Children; GP, General Population; KK, Kato-Katz test; POC-CCA, Point-of-Care platform for detection of cathodic circulating antigen; LCA, Latent Class Analysis;

¶ KK duplicate (Two slides/sample);

* Number of tested samples;

** *Combined 1(parasitological tests): KK, Saline Gradient and the Miracidia Hatch test served as combined reference test*.

**Table 3:** Prevalence and sensitivity values determined by antigen detection assays in high, moderate and low endemicity areas.

**Endemic Area^£^** **(Prevalence)**	**Method** **(Antigen detection assay; biological sample)**	**Reference**	**Study population** **(N^**°**^)**	**New test prevalence/positivity^¶^ %** **(KK prevalence)**	**Reference test**	**Sensitivity %** **(CI 95%)**
Moderate-high	POC-CCA Urine1	Coulibaly et al. (26)	242 SC	64.5 (23.1)	KK^α^	69.7 t –ve (60.7–77.8)
						89.1 t+ve (81.2–93.5)
	POC-CCA Urine1	Erko et al. (39)	620 SC	65.9 (43,1)	K-K^#^	93.0 (90.2–95.7)
					Combined1	89.9 (87.1–92.6)
	POC-CCA Urine1	Al-Shehri et al. (32)	258 SC	14–100 (44,1)	LCA	99.1 (97.3–100)
	POC-CCA Urine1	Lodh et al. (34)	100 GP	60 (51)	Combined 2	67 (56–77)
Low-moderate	POC-CCA Urine 1	Bezerra et al. (40)	258 GP	3.9 (1.2)	–	–
	POC-CCA Urine 1	Ferreira et al. (29)	300 GP	27.3 (6.0)	LCA	68.1
					K-K^#^	64.3 (38.8–83.6)
					K-K^£^	57.7 (38.9–74.5)
	POC-CCA Urine 1	Siqueira et al. (41)	163 GP	22.6 (10.6)	K-K^¶^	73.5 (56.9–85.4)
	POC-CCA Urine 1	Lindholz et al. (42)	461 GP	40.6–71.6 (11.9)	LCA	57.4 t –ve (50.0–64.6)
						81.9 t+ve (75.7–87.1)

£ Classified according WHO; POC-CCA, Point-of-Care platform for detection of cathodic circulating antigen; POC-CAA, Point-of-Care platform for detection of anodic circulating antigen; SC, School Children; GP, General Population; KK, Kato-Katz test; LCA, Latent Class Analysis; Urine 1: single – urine CCA; KK prevalence test was determined by using two slides/sample (duplicate); Sensitivity calculation and reference test:

# KK single slide/sample;^£^K-K AS: any positive sample;

¶ KK duplicate (Two slides/sample, 1 sample);

α KK quadruplicate (Two slides/sample, 2 samples);

* *Number of tested samples; Combined1: 6 KK and 3 POC-CCA served as combined reference test; Combined2: KK and PCR; t-ve: trace negative; t+ ve: trace positive*.

Evaluation of drug response by parasitological methods at the community level in LEAs is unreliable for a long time (9, 13, 28–30, 43). More than a decade has passed since studies pointed out the progressive loss of sensitivity in parasitological methods with decreasing prevalence and an associated difficult in measuring treatment efficacy (44). Teles et al. (45) demonstrated a progressive reduction of schistosomiasis prevalence from 3.4 to 1.8% in the 3-year observation period in an LEA. The prevalence rate decreased sharply after treatment. However, some egg-positive cases remained detectable after treatment, with no change in parasite load. Since only an egg-based assay was used, non-egg excretors were not diagnosed and treated. Diagnosis based on egg detection does not reveal residual infections with low- or no-egg excretion, only those infections with a persistent parasite burden (2). Other studies presented similar data, demonstrating the failure to distinguish the status of control vs. elimination of schistosomiasis in transmission areas based exclusively on egg detection, as shown in Table 1.

Decreased test sensitivity jeopardizes surveillance of endemic areas by compromising the mapping and monitoring hotspots of transmission and accurately assessing the impact of interventions like preventive chemotherapy use, sanitation and health education (9). However, the limitations of conventional methods are not restricted to transmission areas. At institutional settings in non-endemic areas, both diagnosis and clinical management of individuals with a history of exposure to contaminated water could be a problem (43). For example, the diagnosis of acute schistosomiasis during the prepatent period, with low parasite burden or an absence of egg excretion during chronic infection in travelers, immigrants and refugees often leads to misdiagnoses (23). Low-cost alternatives, like egg enrichment methods, such as Helmintex^TM^, have potential to improve egg detection. By using magnetic beads to trap eggs in a magnetic field, Helmintex^TM^ enhances microscopic efficacy. It is a very sensitive (100%) technique to detect *Schistosoma* infection in individuals with as few as 1.3 epg of feces (46). The data suggest that the method could be a reliable alternative to enhance the diagnostic performance of egg-detection assays in LEAs and for the diagnosis of infections with low worm load (25). However, it is a laborious test, and in areas of co-endemicity it may not distinguish *Schistosoma* species. Also, species-specific differences in eggs' ability to bind to the magnetic beads must be determined for its use in urinary schistosomiasis and “new” infections induced by hybrid species (9). More extensive studies should be conducted.

Also, during chronic infections, low parasite burden can remain unnoticed in asymptomatic individuals. However, in individuals with advanced disease and/or atypical presentations, parasitological tests and even tissue sample analysis can miss active infections (43). As a result, schistosomiasis diagnosis by egg detection in particular groups—like travelers, immigrants and refugees—is underdiagnosed and clinical management becomes restricted.

The use of conventional tests for the diagnosis of schistosomiasis in endemic and non-endemic areas seems to have reached a crossroad; reliable new diagnostic approaches are urgently needed.

## Non-Traditional Diagnostic Approaches Currently Available May Change Schistosomiasis Management Before and After Treatment in LEAs and Non-Endemic Areas

Although egg detection remains as a reference for the diagnosis of schistosomiasis at community and individual levels, diagnostic assays for DNA and antigen detection can overcome the limitations of parasitological tests. DNA detection assays are mostly in-house tests, although some are becoming commercially available (10). PCR-based assays detect DNA of *Schistosoma* in stool, serum, plasma, urine, and tissue, demonstrating high sensitivity and specificity (Table 2) (7, 36, 47).

Studies carried out in high, moderate and low endemicity areas suggest that DNA-based assays are reliable tools for schistosomiasis diagnosis and for monitoring treatment response (32–38, 47). In the field, PCR-based assays are more sensitive than parasitological tests regardless of the area endemicity (Table 2). PCR-based methods demonstrated higher sensitivities (80–100%), and—in LEAs— resulted in prevalence values that were higher than those of the K-K technique (Table 2). Amplification of the *S. mansoni* 121-bp tandem-repeat sequence by a conventional PCR test in one fecal sample showed a higher prevalence than K-K analysis of three stool samples from individuals living in LEAs (Table 2). By using real-time PCR targeting the COX-1 target region, Cavalcanti et al. (30) demonstrated that *Schistosomiasis mansoni* prevalence was 12.96% compared to K-K estimated prevalence of 7.4% in a low endemicity setting (Table 2). DNA amplification was demonstrated in up to 100% of the egg excretors and 85.7–94.7% of serologically-reactive individuals. Loop-mediated isothermal amplification (LAMP) is a cheaper and simpler version of a molecular detection assay. LAMP is user-friendly and does not require a thermocycler (10). In LEAs, LAMP showed superior performance compared to K-K (Table 2). There is growing evidence that LAMP might be an affordable diagnostic approach in low-income settings. A small study in Brazil indicated that LAMP detected almost 10 times more active infections of schistosomiasis in humans than a duplicate K-K smear. The test detected 92.31% of the egg excretors and 24.83% of K-K negative individuals living in a LEA (37).

Robust data demonstrated that PCR-based methods are also reliable approaches to monitoring response to treatment (Table 2). In areas of high prevalence and infection intensity, at 2 months after PZQ treatment, single real-time PCR detected 69, 3% of *S. haematobium* infected children vs. 22.8% demonstrated by microscopy (14). After 18 month follow up, real-time PCR diagnosed 78.9% of positive samples in contrast to 63.2 to 68.1% detected by microscopy. In LEAs, persistent DNA amplification can be demonstrated in up to 50% of previously treated individuals with schistosomiasis mansoni 6 months after a single round of PZQ therapy, while no K-K positivity was found [Supplementary Material, (30)]. In LEAs, “false” rapid clearance of egg excretion until 6 months post-therapy may occur while amplification of DNA persists (30). However, during a 2-year long follow up, results showed that both egg and DNA detection had similar performance in a study population of pretreated individuals [Supplementary Material, (30)].

By using PCR-based assays to assess PZQ cure rates, rt-PCR demonstrated that 24% and 18.2% of the treated individuals with one and two rounds of therapy, respectively, remained positive after treatment (30). The results suggest that persistent DNA amplification after PZQ use may be a more suitable indicator of cure.

PCR-based assays detect *Schistosoma* DNA in stool, serum, plasma, urine and tissue, demonstrating high sensitivity and specificity (Table 2) (38, 48). DNA detection assays are also useful in cases of co-infection with different *Schistosoma* species and soil-transmitted helminths (STHs); they have potential use in both STH and schistosomiasis control programs (38, 49–51). Multiplex diagnostic platforms allow the diagnosis of multiple STHs and *Schistosoma*, and may be a cheaper alternative for low-income areas (49).

Egg-negative infections missed by conventional methods can also be revealed by DNA detection assays in travelers, immigrants and refugees leaving non-endemic areas (48). Based on a small case series, real-time PCR for DNA detection in serum was shown to surpass K-K and serology and improve the diagnosis of acute infections in egg-negative and/or serologically inconclusive individuals (52). One crucial pitfall is the amplification of certain *Schistosoma* target genes. In the same case series, the authors discuss how a repetitive 121-bp DNA fragment represents a little more than 10% of the *S.mansoni* genome. Its use as a target sequence in real-time PCR might reduce test sensitivity in infections other than *S. mansoni*, such as *S. haematobium* and *S. japonicum*. In situations of co-endemicity or hybrid introduction, the test could have lower performance.

## Antigen Detection Might Play a New Role in Schistosomiasis Diagnosis

Lately, different assays for the detection of *Schistosoma* biological sub-products became investigated as reliable alternative to ova detection. Antigen detection was made possible through the development of immunodiagnostic assays in the 1980s (53, 54). Enzymatic (ELISA) assays to detect *Schistosoma* gut-associated polysaccharides—CCA and CAA—turned out to be a promising diagnostic approach (10, 55). In the 1990s, study in area of high endemicity showed that immunodiagnostic assays (e.g., ELISA) for detection of CAA and CCA in serum and urine demonstrated active *S. mansoni* infection in 94, 83, and 95% of the populations studied, of which 91% were positive by stool exam. Data showed that CCA and CAA levels correlated with egg counts, although these antigens could also be detected in egg-negative individuals. ELISA for CCA and CAA also demonstrated higher sensitivities than a single stool exam (56). But when testing the applicability of CAA and CCA detection in a low endemicity area, results showed that sensitivities for CAA and CCA using ELISA were 23% and 17%, in serum and 3 and 28% in urine samples, respectively. In contrast, egg detection was positive in 29% of the examined cases in the same area. Based on this, the study suggested that the detection of CCA in urine and CAA in serum might be a suitable diagnostic approach for schistosomiasis, and may be used in conjunction with the parasitological tests to determine the prevalence and intensity of *Schistosoma* infection in LEAs (57). At this time, CCA and CAA detection assays use were limited. But, a huge step was taken after lateral flow (LF) based assays became available by changing the scenario of schistosomiasis diagnosis (58, 59).

In the era of rapid tests for the diagnosis of many infectious diseases, LF-based assays for CAA and CCA detection have the potential to revolutionize the field of schistosomiasis diagnosis. *Schistosoma* antigens are detectable in serum and urine samples. CAA-detection based assay uses luminescent quantitative up-converting phosphor (UCP) reporter particles (UCP-LF). But, the assay is not available in the market currently. The point-of-care (POC) test for CCA detection has a commercial version (10, 60). Detection of *Schistosoma* species is highly variable. POC-CCA, e.g., is more reliable for *S. mansoni* than for *S.haematobium* detection, although it can also detect *S. japonicum* active infections (59). By contrast, UCP-LF CAA may be a promising diagnostic tool in urinary schistosomiasis. A study carried out in Zanzibar of 2,067 randomly selected school children from 16 schools evaluated UCP-LF CAA test accuracy in areas with prevalences ranging from <2 to 10%. By using latent Bayesian analyses (LBA), sensitivity of UCP-LF CAA was estimated to be 97% (95% CI: 91–100%), and suggested a 14% prevalence. Results showed that high sensitivity and reasonable specificity values assured that the UCP-LF CAA test could be a reliable approach for diagnosis of urogenital schistosomiasis in LEAs attempting complete elimination (61). In non-endemic areas, results from a study of 81 serology-positive individuals demonstrated that UCP-LF CAA confirmed active infections in 56/81 individuals exposed to contaminated waters (62). Also, acute infection was diagnosed before oviposition, since CAA levels were detectable 4 weeks after exposure. CAA levels become measurable before detection of *Schistosoma*-specific antibodies. Moreover, the study also detected different CAA concentrations in the sera of travelers and migrants, with higher CAA levels demonstrated in migrant serum samples. Post-treatment, a sharp decrease of CAA levels was demonstrated. The results suggest that a UCP-LF CAA serum assay would be a reliable test for diagnosing light infections in non-endemic settings (62).

Field studies presented promising results, and the diagnosis of active schistosomiasis was made easy by the commercial version of CCA manufactured by Rapid Test Diagnosis (Pretoria, South Africa). In high and moderate endemic areas, POC-CCA showed higher accuracy than the K-K technique (26, 39). The results also indicated that the intensity of the POC-CCA reaction correlated with the number of eggs (26). By contrast, results also showed that antigen daily variability was frequently observed in school children as well as antigen persistence in the absence of egg excretion after PZQ treatment (26). In Table 3, POC-CCA sensitivity ranged from 67 to 99.1% when applied in areas of high and moderate endemicity. However, studies performed in populations with light infections and/or LEAs did not experience the unquestionable success of the high and moderate endemicity areas (63). The accuracy of POC-CCA is reduced as a result of an increased number of false positive reactions (Table 3) (20). Mostly, the presence of very weak reactions—called trace positives—overestimates the estimation of active infections. In LEAs, the trace reactivity in POC-CCA is poorly associated with positivity in other confirmatory tests (64). However, it remains undetermined whether the data showing trace results may represent accurate positive reactions and instead should confirm active schistosomiasis (65).

In non-endemic areas, antigen-detection assays' accuracy must be further investigated. In a retrospective study of immigrants and refugees, CCA showed low sensitivity when compared to microscopy, ELISA, Western blot and immune-chromatographic tests (66). Although some authors agree with the usefulness of POC-CCA as a tool for detecting active infection in special populations like refugees, use of the test after treatment follow up is still disappointing in this group (67).

## Discussion: About the Controversies, Current Research Gaps and Potential Future Developments in the Field

The main question in an era of control and elimination of *Schistosoma* infection in a transmission areas is: can the diagnosis of schistosomiasis in endemic settings rely on one test? Although the K-K technique has been applied over the years, several adaptations of the method did not improve its skill in diagnosing schistosomiasis. At the moment, ideal tests are inexistent, and gold standard or reference tests in schistosomiasis diagnosis are missing in LEAs. Limitations of the K-K technique include loss of test sensitivity, low reproducibility, and it is a time-consuming technique even though it has a lower cost compared to molecular tests. Applicability of the K-K in LEAs has become practically useless since a low parasite burden is often hidden within individuals with no egg excretion. Also, K-K performance for diagnosis of multiple parasitic infections in co-endemic areas becomes poor when parasitological tests are used. In institutional settings, primary healthcare of travelers, immigrants, and refugees is compromised by stool exam low performance in light infections. As a marker of drug response or complete cure, the parasitological tests may underestimate the number of treatment failures to PZQ (non-responders) among individuals living in areas of persistent transmission (11).

Since diagnosis limited to conventional tests compromises the detection of active schistosomiasis, it is time to consider new approaches. In the case of *S.mansoni*, the substitution of DNA or antigen detection assays for stool exam has been considered before (68). Both of the molecular detection assays uncover active infections in both egg excretors and non-excretors. DNA- and antigen-based assays are gradually showing increased potential as markers of active infection in endemic and non-endemic areas. DNA detection assays seem to be an eligible tool for assessing response to therapy in transmission and non-endemic areas. However, superior performance can be seen when molecular approaches are used as a single confirmatory test in both endemic and non-endemic areas. Inferior performances by DNA amplification techniques may be the result of a poor choice of target gene amplification regions for the species under investigation. Also, its universality is debatable under this argument: the costs of DNA-based assays in low-income settings can be 10 times more expensive than the alternatives (10). One possible solution to drop the costs could be the use of multiplex platforms by the Global Polio Laboratory Network (GPLN) in areas of STH and SCH co-endemicity (49). The GPLN already performs molecular diagnosis for poliovirus and other enterovirus infections in 16 labs across Africa. By using multiplex platforms for the diagnosis of STH and SCH, detection of multiple parasitic active infections might be improved while assessment of drug response in transmission areas is kept to a minimal cost. Antigen detection assays are quickly becoming the new reference test of preference in areas of schistosomiasis transmission. However, standardization of the assays, as well as increased sensitivity and specificity and increased availability of multiple commercially affordable tests, are essential issues to be addressed by national schistosomiasis control programs.

Overall, diagnostic tests unveil different aspects of the *Schistosoma* biology. Detection of *Schistosoma* and its subproducts may not overlap during the parasite cell cycle. Distinct from egg detection, the kinetics of *Schistosoma* antigens and DNA are still poorly established during *Schistosoma* maturity. In budget-limited national programs, it is fundamental to have an accurate, reliable, easy-to-use, and cheap diagnostic tool available to diagnosis schistosomiasis. However, the use of a single tool might restrict the management of *Schistosoma* infections at community and institutional levels. Hence, adoption of multiple diagnostic approaches should be considered in areas of low endemicity.

## Conclusion

Evidence of the necessity of settling the new options for the detection of active schistosomiasis is overwhelming. However, it appears more research must be conducted before a final decision is made regarding the total substitution of the conventional tests such as parasitological assays.

## Author Contributions

MC and JP: the conception and design of the work. MC and AC: performed the literature review. MC and JP: contributed to draft manuscript editing/reviewing. All authors contributed to the revisions and approved the final version of the manuscript.

### Conflict of Interest Statement

The authors declare that the research was conducted in the absence of any commercial or financial relationships that could be construed as a potential conflict of interest.
